# Connexin-Mediated Functional and Metabolic Coupling Between Astrocytes and Neurons

**DOI:** 10.3389/fnmol.2018.00118

**Published:** 2018-04-11

**Authors:** Lady C. Mayorquin, Andrea V. Rodriguez, Jhon-Jairo Sutachan, Sonia L. Albarracín

**Affiliations:** Departamento de Nutrición y Bioquímica, Facultad de Ciencias, Pontificia Universidad Javeriana, Bogotá, Colombia

**Keywords:** gliotransmission, connexins, synaptic plasticity, neurons, astrocytes, neurodegenerative disease, hypoxia, ischemia

## Abstract

The central nervous system (CNS) requires sophisticated regulation of neuronal activity. This modulation is partly accomplished by non-neuronal cells, characterized by the presence of transmembrane gap junctions (GJs) and hemichannels (HCs). This allows small molecule diffusion to guarantee neuronal synaptic activity and plasticity. Astrocytes are metabolically and functionally coupled to neurons by the uptake, binding and recycling of neurotransmitters. In addition, astrocytes release metabolites, such as glutamate, glutamine, D-serine, adenosine triphosphate (ATP) and lactate, regulating synaptic activity and plasticity by pre- and postsynaptic mechanisms. Uncoupling neuroglial communication leads to alterations in synaptic transmission that can be detrimental to neuronal circuit function and behavior. Therefore, understanding the pathways and mechanisms involved in this intercellular communication is fundamental for the search of new targets that can be used for several neurological disease treatments. This review will focus on molecular mechanisms mediating physiological and pathological coupling between astrocytes and neurons through GJs and HCs.

## Introduction

Classical studies carried out by the end of 19th and during the 20th century classified astrocytes mainly based on their differences in morphology and localization into two different populations, protoplasmic and fibrous (Ramón y Cajal, 1909). However, recent studies have revealed a more intricate and complex level of astrocyte organization in the central nervous system (CNS). Specialized astrocytes and stem cells with astrocyte features have been found in the cerebellum and retina (Bergmann glia and Müller cells) and neurogenic niches (Ben Haim and Rowitch, 2017). Likewise, these astrocyte populations are characterized by different marker expression. Astrocytes located in the white matter and astrocytic stem cells express high levels of glial fibrillary acid protein (GFAP). In contrast, protoplasmic astrocytes exhibit low expression of GFAP but high levels of the potassium channel Kir4.1 (Ben Haim and Rowitch, 2017). Although, it remains unclear why astrocytes achieve such diversity; this could be an adaptive mechanism to the activity of specific neuronal networks.

Due to their intimate association with neurons and blood vessels cells, astrocytes play a metabolic, structural and regulatory role in the CNS. For instance, astrocytes supply energetic substrates (glucose, lactate, citrate, and glutamine) to neurons, participate in the turnover of neurotransmitters such as glutamate and gamma-aminobutyric acid (GABA; Barres, 2008; Sofroniew and Vinters, 2010), and regulate the blood-brain barrier (BBB) permeability, and the homeostasis of ions and pH (De Bock et al., 2017). In addition, astrocytes can release different growth factors that regulate synapse formation and expression of tight junction proteins (Liebner et al., 2011).

Complex astrocyte endeavors are in part explained by their intercellular communications through gap junctions (GJs). This specialized intercellular communication gives rise to a complex syncytial network that allows diffusion of several essential molecules for signaling and information processing (Robertson, 2013). GJs and hemichannels (HCs) formed by connexins (Cxs) allow small molecule diffusion that induces and regulate functional and metabolic coupling between astrocytes and neurons. Neurotransmitters released by neurons bind to astrocyte membrane-bound receptors, resulting in the production of metabolites, such as inositol 1,4,5 trisphosphate (IP_3_), adenosine triphosphate (ATP), glutamate, lactate and D-serine that diffuse through GJs to impact back on neuronal function (Orellana and Stehberg, 2014). As a case in point, in the gray matter, it has been shown individual protoplasmic astrocytes occupying non-overlapping spatial domains, where each astrocyte can interact with hundreds of dendrites and neuronal cell bodies (Oberheim et al., 2009; Sofroniew and Vinters, 2010). This bidirectional interaction is fundamental for healthy brain function, and impairment in this communication can lead to the development of pathological conditions. This review will focus on cellular and molecular mechanisms that mediate metabolic and functional coupling between astrocytes and neurons, and how Cxs-mediated intercellular communication in astrocytes play a crucial role in this coupling. Additionally, we will review our current understanding of the neuroglial interaction emerging role in some neurological disease development.

## Connexin Expression in the CNS

**Figure 1:**
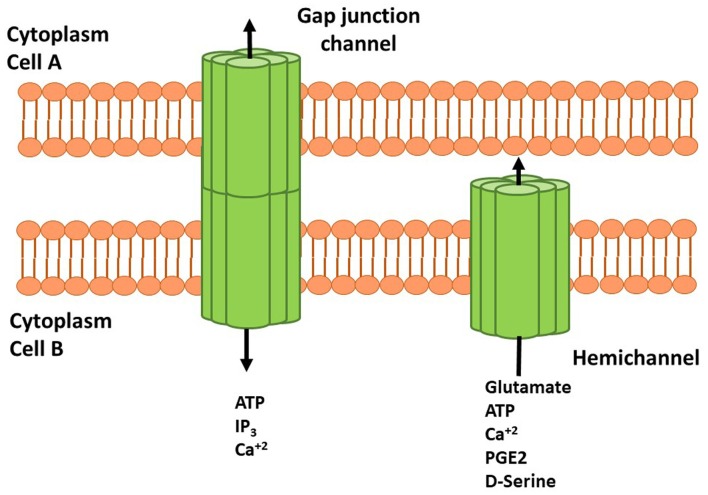
Schematic representation of connexins (Cxs), hemichannels (HCs) and gap junctions (GJs) in cell membranes. HCs are transmembrane proteins composed of six connexin subunits that allow flow of several molecules and gliotransmitters from astrocyte to the extracellular space. Adenosine Triphosphate (ATP), glutamate, D-serine and prostaglandin (PG) E-2 can interact with their receptors and induce signaling cascades. Docking between two HCs or connexins forms GJ channels that allow the cell to cell communication mediated by ATP, inositol 1,4,5 trisphosphate (IP3) and Ca^2+^. HCs and GJs activity modulate neuronal synaptic activity and plasticity.

Cxs are a family of transmembrane proteins composed of six subunits forming homomeric or heteromeric HCs that participate in small molecule release (up to 1.5 kDa) directly to the extracellular space. HCs also dock with other HCs or connexons of adjacent cell membranes to establish GJs (White and Bruzzone, 1996; Figure 1). At the structural level, Cxs have four transmembrane domains and one intracellular domain involved in channel regulation by post-translational modifications (i.e., phosphorylations) or interactions with cytosolic proteins (Márquez-Rosado et al., 2012; Matsuuchi and Naus, 2013; Figure 1). Of the 21 Cxs isoforms so far described, 11 have been detected in the CNS. Cxs in astrocytes, oligodendrocytes, microglia and neurons are characterized by developmental state, region and cell-type specific isoform expression, suggesting a critical role of these proteins in regulation and maintenance of several CNS functions (Lapato and Tiwari-Woodruff, 2018).

Cxs not only allow ions, small metabolites, or second messengers flow but also mediate or regulate other processes in the CNS. For example, Cx43 controls migration and positioning of excitatory and cortical interneurons during brain development (Elias et al., 2010; Qi et al., 2016). Cxs are also involved in neurogenesis; while Cx43 promotes survival of newborn neurons in the adult mouse hippocampus; Cx30 limits their proliferation and survival (Liebmann et al., 2013). Magnotti et al. (2011) have reported Cx42 and Cx43 are required for astrocyte survival in white matter by mechanisms dependent on Cxs specific expression in both astrocytes and oligodendrocytes. Therefore, differential expression and spatial distribution of Cxs are important determinants of their functions in the CNS.

In the mature brain, astrocytes express high levels of Cx30 and Cx43 and low Cx26 levels. Possibly this expression profile is responsible for determining the autocrine and paracrine signaling interaction that mediates glial and neuroglial communications (Lapato et al., 2017). GJs in astrocytes facilitate the formation of a functional syncytium, promoting ion and neurotransmitter removal released during neuronal activity, and allow propagation of Ca^2+^ waves between astrocytes (Takeuchi and Suzumura, 2014). This interglial communication through Cx-based channels, allows astrocytes to sense and integrate local and global synaptic activity and to respond to gliotransmitters that impact synaptic transmission both pre- and postsynaptically (Bazargani and Attwell, 2016). Hence, Cxs and gliotransmitters are essential players in neuronal activity regulation, behavior, and homeostatic maintenance of brain functions (Volterra and Meldolesi, 2005).

## Connexin-Dependent Regulation of Synaptic Transmission and Plasticity

**Figure 2:**
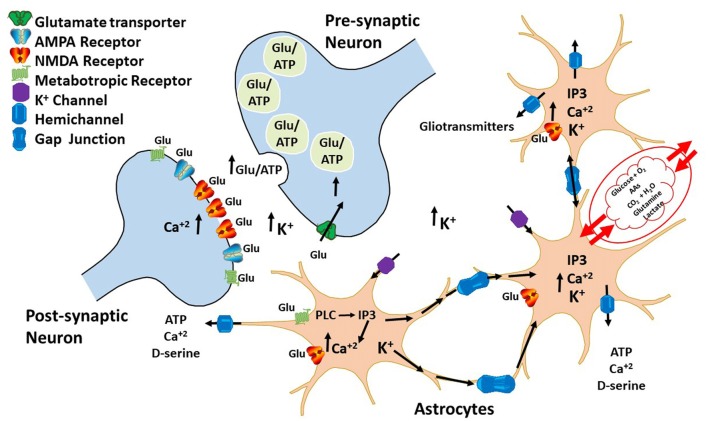
Bidirectional communication between astrocytes and neurons. During neuronal firing, neurons release neurotransmitters that bind to G-coupledreceptors expressed on the surface of astrocytes. The activation of these receptors leads to an increase in IP_3_ and intracellular calcium ions that diffuses through HCs to other astrocytes, inducing gliotransmitter release, such as ATP and glutamate. These gliotransmitters then regulate both glutamatergic and GABAergic neurotransmission by acting either pre- or post-synaptically.

Astrocytes are unable to generate action potentials; however, they can raise intracellular calcium concentrations ([Ca^2+^]i) that spread from cell to cell. Presence of astrocytic calcium ions mobilizations suggests that glial cells may have some excitability and neuromodulator activities (Bezzi and Volterra, 2001; Volterra and Meldolesi, 2005; Dallérac et al., 2013). During intense neuronal firing, glutamate and GABA release induce elevations in glial cells [Ca^2+^]i (Cornell-Bell et al., 1990; Kang et al., 1998). This in turn causes a Ca^2+^-dependent release of molecules that impact on neuronal excitability and synaptic transmission and plasticity (Bazargani and Attwell, 2016). Molecules released by astrocytes upon [Ca^2+^]i increase led to gliotransmitters discovery, which regulates communication between astrocytes and neurons. Hence, it is now accepted that astrocytes work along with neurons through a feedback mechanism, in which neuronal activity induces astrocytes to regulate synaptic transmission and plasticity by releasing gliotransmitters (Figure 2).

According to this paradigm neurons convey information to astrocytes mostly through neurotransmitter release. In this sense, different studies have shown hippocampal CA1 *stratum radiatum* astrocytes respond with an increase in [Ca^2+^]i when activated by ligands. Among the many receptors expressed in astroglial cells are purinergic (Duffy and MacVicar, 1995; Pasti et al., 1997; Porter and McCarthy, 1997; Verkhratsky et al., 1998; Shelton and McCarthy, 1999, 2000; Bowser and Khakh, 2007; Fiacco et al., 2007; Navarrete and Araque, 2008), adrenergic (Duffy and MacVicar, 1995), glutamatergic (Shelton and McCarthy, 1999; Fiacco et al., 2007), and GABAergic. *In vitro* studies have shown glutamate-induced astrocyte [Ca^2+^]i increase through ionotropic (NMDA and AMPA) and metabotropic glutamate receptor (mGLURs) activation (Zhang et al., 2003; Hu et al., 2004). Interestingly, *in vivo* experiments have revealed this [Ca^2+^]i elevation occurs only in some regions of the brain cortex (Pasti et al., 1997; Volterra et al., 2014). A mechanism illustrating *in vivo* cortical astrocyte differential response to glutamate has not been fully established. However, it is thought that some regulatory mechanisms may be conditioning the response of these astrocytes. A recent study revealed hippocampal astrocytes discriminate between neurotransmitters released by different axonal pathways (Perea and Araque, 2005). CA1 astrocytes in the hippocampus respond to glutamate released from CA3 neurons (Schaffer collateral, SC) and to acetylcholine, but not to glutamate released by CA1 neurons (Alveus Terminalis, AT). A plausible explanation for this phenomenon is glutamate protein sensors (receptors and transporters) are differentially localized in these astrocytes. Hence, CA1 astrocytes respond to glutamate released by SC axons, because sensors are expressed in astrocytic processes that are close to these fibers. Nevertheless, the fact that a same population of astrocytes can respond specifically to different neurotransmitters, also suggests astrocytes have intrinsic properties that allow them to discriminate and integrate synapse activity (Perea and Araque, 2005).

Neurotransmitters mainly impact astrocytic [Ca^2+^]_i_ by receptor activation. However, it has also been shown astrocytes express transporters that also regulate synaptic transmission through neurotransmitter uptake (Boddum et al., 2016). GABA is the primary inhibitory neurotransmitter in the brain. It exerts its function by either stimulating chloride ions influx or potassium ions efflux by activating ionotropic and metabotropic GABA receptors (Watanabe et al., 2002). Astrocytes express the GABA transporter GAT-3, whose function is to clear excessive GABA from the synaptic cleft (Ribak et al., 1996). A recent finding suggests GABA uptake throughout GAT-3 can cause inhibition of presynaptic neuronal glutamate release by ATP/adenosine released from astrocytes (Boddum et al., 2016). Different to the canonical pathway in which neurotransmitters directly induce a rise in [Ca^2+^]i, GABA increases astrocyte excitability by movement of sodium ions to the intracellular milieu, resulting in calcium ion influx through Na^+^/Ca^2+^ exchanger. The GABA-dependent Ca^2+^ increase drives ATP/adenosine release that diffuses to excitatory presynaptic terminals, where it inhibits glutamate release and regulates heterosynaptic depression (Boddum et al., 2016).

Cxs have also been implicated in neuroglial communication modulation by gliotransmitter release, impacting pre- and postsynaptic terminals. In prefrontal cortex (PFC), inhibition of Cx43 HCs by mimetic peptide Gap26 reduces the amplitude of postsynaptic excitatory currents mediated by NMDA receptors and impairs long-term potentiation (LTP) induced by high-frequency stimulation (Meunier et al., 2017). In this area, astrocytic [Ca^2+^]i raised by an influx of this ion through Cx43 HCs stimulates D-serine release, a gliotransmitter and co-agonist of NMDA receptors. Hence, D-serine released from astrocytes also through Cx43 HCs in conjunction with glutamate induces NMDA receptor activation, a process that is important for AMPA receptor traffic and stability during LTP (Meunier et al., 2017). These results strongly suggest Cx43 HCs activity in astrocytes is a critical postsynaptic determinant of LTP in the PFC, by regulating NMDA receptor co-activation site occupancy by D-Serine (Henneberger et al., 2010). This effect is apparently independent of astrocyte intercellular communication achieved through GJ channels. Studies using Cx43 conditional knockout mice have revealed a presynaptic regulation of basal excitatory synaptic transmission by Cx (Chever et al., 2014a,b). Deletion of Cx43 in astrocytes induces a decrease in basal excitatory synaptic transmission that is coupled to changes in cell volume, without affecting intrinsic and passive membrane properties of astrocytes and activation of NMDA and AMPA receptors in neurons. Although the mechanism is still unclear, this deficiency in synaptic transmission may be explained by Cx metabolic coupling role between astrocytes and neurons. Astrocyte metabolites such as glutamate, glucose, lactate and glutamine permeate through Cx43 HCs and are taken up by neurons to supply metabolic substrates. It also provides glutamate precursors to refill synaptic vesicles that support excitatory synaptic transmission. Consequently, the observed decrease on basal synaptic excitatory neurotransmission in Cx43 KO mice, can be the result of a glutamate diffusion impartment in astrocytes that lead to glutamine-glutamate cycle failure, operating between astrocytes and neurons (more details in the following section; Bak et al., 2006; Chever et al., 2014b). This cycle is essential for neurons to provide glutamine utilized for glutamate and GABA synthesis at the presynaptic terminal.

Astrocytes like neurons have complex morphologies characterized by the presence of processes that protrude into synapses. For synapses to work efficiently, astrocytes and neurons should have developed mechanisms controlling astrocytic processes extension into synapses, limiting astrocyte territories in the synaptic cleft, which is essential to re-uptake neurotransmitters without affecting synaptic transmission. Cx30 knockout mice show a decreased excitatory neurotransmission and impaired synaptic plasticity (Pannasch et al., 2014). These deficiencies are accompanied by low glutamate levels, increased currents throughout GLT transporters, and significant changes in astrocytic processes extension that insert more in-depth into the synaptic cleft. The observed reduction in synaptic strength in Cx30 KO mice can be the result of an increase in glutamate clearance. This novel role of Cx30 is independent of its channel function, but it is determined by its C-terminal domain interaction with cellular elements that regulate adhesion and migration processes, limiting the extension of astrocytes in the synapse (Pannasch et al., 2014). Similar results have been found in Cx30^−/−^Cx43^−/−^ mice, in which Cxs absence leads to glutamate and K^+^ ions clearance impairments, changes in astrocyte volume, and increases in glutamate and GABA release. To maintain a regular firing without gain modifications, due to abnormal glutamate and GABA levels, Cxs alter AMPA receptor expression in the postsynaptic terminal, inducing homeostatic plasticity (Pannasch et al., 2011). Therefore, the syncytial network established by astrocytes through Cx30 and Cx43 expression is essential for regulating basal synaptic transmission and plasticity by mechanisms that involve neuronal excitability (K^+^ buffering), neurotransmitter release and uptake (Glutamate and GABA), and receptor stability (homeostatic plasticity).

## Astrocytic [Ca^2+^]i Increase Is Coupled With a Gliotransmitter Release Through Cx HCs

Binding of synaptic neurotransmitters to high-affinity G protein-coupled receptors (GPCRs) in astrocytes starts a cascade of events that stimulate membrane phospholipid hydrolysis to inositol triphosphate (IP_3_) and diacylglycerol (DAG; Volterra and Meldolesi, 2005; De Pittà et al., 2009). Diffusion of IP_3_ then induces Ca^2+^ efflux from the endoplasmic reticulum, raising astrocyte Ca^2+^ concentration that drives gliotransmitter release. Although mechanisms that associate [Ca^2+^]i increases to gliotransmitter release in astrocytes have not been fully established, is believed these bioactive molecules are released by Ca^2+^-dependent vesicular exocytosis (as in neurons), and non-dependent vesicular release. Astrocytes express Synaptotagmin IV (a Ca^2+^ sensor expressed in synaptic vesicles) and SNARE type proteins. Synaptotagmin IV knockdown experiments have revealed this Ca^2+^ sensor is involved in glutamate release from astrocytes (Zhang et al., 2004). Similarly, astrocytes express synaptobrevin II and cellubrevin, two proteins of the SNARE complex that regulate glutamate and neuropeptide-Y release. Remarkably, these two gliotransmitters are not only packed in different vesicles, but they also exert different effects in synaptic transmission (Schwarz et al., 2017).

Non-vesicular gliotransmitter release is achieved through reversible uptake carriers (Rossi et al., 2000), volume-activated anion channels (Rudkouskaya et al., 2008), purinergic receptors (Suadicani et al., 2006), and Cx and pannexin HCs (Panx HCs; Jiang et al., 2011; Orellana et al., 2011). The mechanisms by which astrocyte intracellular Ca^2+^ changes are coupled to gliotransmitters release through Cx and Panx HCs are not fully understood. However, recent findings suggest Ca^2+^ levels regulate Cx HCs opening probability. For example, Cx32 HCs have a Ca^2+^ binding site rich in aspartate residues that sense and decode [Ca^2+^]e changes in channel gating (Gómez-Hernández et al., 2003). Moreover, Cx32 HC opening is triggered by changes in [Ca^2+^]i in the range of 200–1000 nM. The presence of calmodulin binding sites in Cx32 and Cx43 HCs and the fact that calmodulin inhibitors block connexin-ATP release, suggest this Ca^2+^ sensor is part of the Ca^2+^ pathway utilized to regulate Cx HCs gating (De Vuyst et al., 2006; Lurtz and Louis, 2007).

Other studies using mouse epidermal cells have determined GJ intracellular communication is mediated by a Ca^2+^-dependent regulation of E-cadherin cell adhesion molecule (Jongen et al., 1991). Although this mechanism has not been evaluated in astrocytes, the above results suggest interactions between Cxs and cell adhesion molecules mediated by Ca^2+^ increases can be essential for astrocyte syncytium stability during neuronal activity.

Paracrine ATP action can complicate Ca^2+^ signaling in astrocytes. Extracellular ATP signaling is crucial in the CNS, and its interaction with P2-purinoceptors (P2Rs) has critical neuromodulator functions (Tozaki-Saitoh et al., 2011; Illes et al., 2012). P2YRs are metabotropic receptors coupled with different G-proteins that mediate phospholipase C (PLC) signaling, associating IP_3_ diffusion to Ca^2+^ mobilization via GJs (Cotrina et al., 1998; Illes et al., 2012). Hence, ATP- Ca^2+^ mobilization in astrocytes works as a feedback mechanism, regulating Ca^2+^ diffusion in astrocytes that allow local synaptic transmission to influence synaptic activity over a large population of neurons.

## Metabolic Coupling Between Neurons and Glial Cells

**Figure 3:**
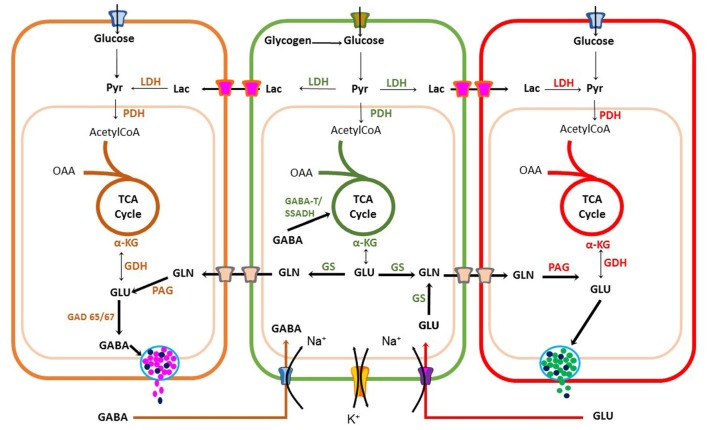
Metabolic coupling between neurons and astrocytes during synaptic transmission. Glutamatergic and GABAergic neurons have an oxidative metabolism that depends mainly on glucose. Neurons use energy for restoring the ionic balance after action potential firing and for neurotransmitter synthesis. Astrocytes also play a significant role in recycling and inactivation of gamma-aminobutyric acid (GABA) and glutamate neurotransmitters through glutamate-GABA-glutamine cycle, also important for ammonium ion buffering. Astrocytes can also produce lactate upon increase in neuronal activity to sustain high firing rate. Pyr, Pyruvate; LDH, Lactate Dehydrogenase; OAA, Oxaloacetic acid; TCA, tricarboxylic cycle acid; αKG, alpha-ketoglutaric acid; GDH, Glutamate dehydrogenase; GLN, Glutamine; PAG, Phosphate-activated glutaminase; GAD, glutamic acid decarboxylase; GS, Glutamine Synthetase.

Astrocyte [Ca^2+^]i increase is also used as a signal to trigger metabolic linkage between neural activity and energy demands to sustain neuronal firing. Coupling is achieved by releasing messengers, such as arachidonic acid (AA) that regulates blood vessel tone or by increasing glucose uptake from blood (Bazargani and Attwell, 2016). This energetic coupling between astrocytes and neurons is also achieved by regulating biosynthesis, degradation and uptake of excitatory and inhibitory neurotransmitters (Figure 3).

Astrocytes express transporters that mediate glucose uptake from adjacent cerebral microvessels through glucose type 1 transporters (GLUT1; Rouach et al., 2008). Glucose is stored as glycogen and then is partially oxidized through the glycolysis pathway to produce pyruvate. However, during intense neuronal activity astrocytes reduce pyruvate to lactate and export this energetic substrate through monocarboxylate transporters 1 or 4 (MCT1-4) to the synaptic cleft, which can be taken-up by neurons via MCT2 transporters. This coupling between cells is known as astrocyte-neuron lactate shuttle (ANLS) hypothesis (Pellerin and Magistretti, 1994). Additionally, astrocytes and neurons can completely oxidize pyruvate in the Krebs cycle (KC). Neuronal aerobic glucose catabolism leads to a net synthesis of alpha-ketoglutarate (α-KG), catalyzed either by glutamate dehydrogenase (GDH) or an amino acid aminotransferase (AAT; Figure 3). As a result, glutamate can be synthesized and used for excitatory synaptic transmission or decarboxylated by a glutamic acid decarboxylase (GAD 65/67) for GABA synthesis in inhibitory presynaptic terminals (Walls et al., 2010, 2011).

A family of five glutamate transporters, called GLAST, GLT-1, EAAC1, EAAT4 and EAAT5 (also known as EAAT1-5), are responsible for maintaining extracellular glutamate concentrations within a range that permits normal excitatory neurotransmission (Tzingounis and Wadiche, 2007). Astrocytes, transform glutamate to glutamine, through a reaction catalyzed by glutamine synthetases (GSs) with an expenditure of 1 ATP (Krebs, 1935). Then, glutamine is released into the extracellular milieu and is taken-up by neurons to synthesize glutamate by a phosphate-activated glutaminase (PAG; Krebs, 1935). Use of glutamate and GABA for glutamine biosynthesis by astrocytes gives origin to the Glutamate-GABA-Glutamine cycle operating between glutamatergic and GABAergic tripartite synapsis (Bak et al., 2006; Figure 3). Therefore, the coupling between excitation and inhibition is a crucial condition required for normal neurotransmission. Synthesis and re-uptake of both glutamate and GABA demand neuroglia compartmentation. Glutamate-GABA-Glutamine cycle by maintaining low neurotransmitter concentrations in the synaptic cleft protects neurons against glutamate excitotoxicity. However, it is an expensive transport process requiring high ATP levels. Each neurotransmitter molecule is co-transported with three sodium ions (Na^+^), requiring Na^+^/K^+^ ATPase activity. Furthermore, in astrocytes, this coupling demands glucose utilization and oxidation (Hertz, 2013; Schousboe et al., 2013a). Hence, to sustain significant glutamate removal from the synaptic cleft, astrocytes must produce enough ATP from different energetic substrates. Another essential function of the Glutamate-GABA-Glutamine cycle is ammonium (NH_4_^+^) transport (Figure 3), which is potentially toxic to neurons and it must be taken up by the surrounding astrocytes. This is a clear example of compartmentation between neurons and astrocytes (Hertz, 2013; Schousboe et al., 2013a,b). Synaptic transmission and its regulation by gliotransmitters is a demanding energy process. It requires metabolic coupling to provide energetic substrates to sustain ATP production for neurotransmitter uptake, ion pumps and guarantee the necessary precursors replenishment for neurotransmitter synthesis to maintain synaptic transmission.

## Role of Connexins in CNS Diseases

As discussed above, astrocytic Ca^2+^-dependent signaling pathways are activated in healthy brain tissue. However, dramatic changes in astrocytic [Ca^2+^]i transient frequencies, duration and amplitude have been reported in epilepsy (Ding et al., 2007), Alzheimer’s disease (AD; Kuchibhotla et al., 2009), and stroke (Rakers et al., 2017). These observations suggest changes occurring in reactive astrocytes could regulate liberation of gliotransmitters, instead of synchronizing normal metabolic coupling that could exacerbate injuries or lead to neurodegeneration. Some authors have proposed these events could be a consequence of a rapid response, seconds to minutes, part of a serial of excitotoxic events that are common to these diseases (Agulhon et al., 2012). For instance, damaged neurons after ischemia decline their aerobic metabolism, decrease ATP production, and accumulate toxic ions and molecules, such as Ca^2+^, K^+^, radical oxygen species (ROS), and nitric oxide (NO). These toxic molecular species can be released through HCs and propagated from damaged cells to healthy cells through GJs (Takeuchi and Suzumura, 2014). Under these conditions, those signals can activate microglia and astrocytes, inducing the release of pro-inflammatory cytokines and chemokines (Orellana et al., 2009). These inflammatory mediators can exert a modulatory effect on astrocyte physiology, causing alteration on neuronal functions that affect mood, behavior and cognitive abilities (Sofroniew, 2014). At the cellular level, pro-inflammatory cytokines reduce the intercellular communication mediated by GJs and increase Cx HCs activity, leading to an increase in astrocytic ATP and glutamate release (Retamal et al., 2007; Orellana et al., 2011). Under this inflammatory scenario, astrocytes not only produce, but they also respond to molecules such as tumor necrosis factor α (TNFα), transforming growth factor β (TGFβ), IL1β, IL6, interferon γ (IFNγ), CCL12, glial derived neurotrophic factor (GDNF), and proteins, such as fibrinogen, thrombin and endothelin-1 (Hamby et al., 2012; Sofroniew, 2014). Other molecules, such as prostaglandins (PG), NO and AA are produced by astrocytes and can induce deleterious effects in neurons (Avila-Muñoz and Arias, 2014). Neurodegenerative diseases like Alzheimer, Parkinson and other pathological conditions, such as ischemia and traumatic brain injury (TBI) will be discussed in the context of Cx HCs activity in response to injury.

## Cxs in Alzheimer’s Disease

AD is the leading prevalent dementia in the world and is considered a neurodegenerative condition characterized by a decline of the cognitive function related to a progressive cortical neuronal loss. Although the primary risk factor is aging, at the molecular level, AD has been associated with extracellular accumulation of beta-amyloid peptide (Aβ) and intracellular neurofibrillary tangles of hyperphosphorylated Tau protein, loss of synaptic connections, and oxidative stress increase leading to neuronal death (Querfurth and LaFerla, 2010). A classical AD study in human brains found increased Cx43 expression in reactive astrocytes near amyloid plaques, activated microglia, and neurons (Nagy et al., 1996). Other studies using two murine models of AD for β-amyloid precursor protein (APP) and presenilin 1 (PS1) have found Cx43, and Cx30 expression was increased in activated astrocytes that were associated with amyloid plaques in the pyramidal cell layer of the hippocampus at 6 months (Mei et al., 2010). Human brain samples with a diagnosis of AD and Parkinson’s disease (PD) have also identified increased Cx43 expression associated with astrocytosis, a process that correlates with the progression of both diseases (Kim I. S. et al., 2016). Moreover, studies evaluating APP/PS1 mice have shown Cx43 overexpression that correlates with gliotransmitter increments release, such as ATP, glutamate, and Ca^2+^, increase in oxidative stress and exacerbated neuronal damage (Yi et al., 2016). Nowadays it is a matter of debate if the increased Cxs expression in AD is part of an adaptive molecular mechanism to restore neuroglia homeostasis communication, or instead, it is an aberrant protein expression that exacerbates cellular conditions and microenvironment favoring disease progress.

## Cxs in Parkinson’s Disease

PD is the second neurodegenerative disorder worldwide. Its main feature is the loss of dopaminergic neurons in the substance nigra pars compacta resulting in decreased dopamine levels, increased oxidative stress, and mitochondrial dysfunction in the striatum. As consequence patients display bradykinesia, rigidity and tremor (Lees et al., 2009). A recent study has shown that overexpression of Cx43 protects SH-SY5Y neuroblastoma cells, against mitochondrial-induced apoptosis by regulating mitochondrial permeability transition pore closing, preservation of mitochondrial membrane potential, and decreasing cytochrome C release (Kim I. S. et al., 2016). *In vitro* and *in vivo* studies using rotenone-induced models have shown increased expression of Cx43 and its phosphorylated form (Kawasaki et al., 2009). However, these changes in Cx43expression levels do not correlate with neuronal survival. Phosphorylation of Cx has been associated with functional GJs assembly, therefore in PD Cx43 phosphorylation could be related to mechanisms that reestablish normal synaptic function and promote neuronal survival.

Other studies using SH SY5Y cells that overexpress wild-type α-synuclein demonstrated those cells were more susceptible to hydrogen peroxide and 6-hydroxydopamine. This increased susceptibility was mediated by a molecular interaction between Cx32 and α-synuclein, suggesting that modulatory mechanisms of GJs can be essential to high sensitivity to neuronal toxins (Sung et al., 2007).

## Cxs in Acute Cerebral Ischemia and Traumatic Brain Injury (TBI)

Anomalous functioning of Cx43 after ischemic insults causes apoptotic cell death. In a cerebral rat model of ischemia, clamping both arteries increased expression of heteromeric Cx40/Cx43 connexons and Cx43 phosphorylated form. This increase in Cx levels was mediated after ischemia by extracellular signal-regulated kinase (ERK) activation (Chen et al., 2017), suggesting heteromeric Cx complexes contribute to brain damage.

In neonatal rat models of hypoxia/ischemia (HI), Cx43 expression increased, and pre-treatment with Gap26 and Gap27 peptides powerfully decreased cerebral infarct volume and improved muscle strength, motor coordination and spatial memory skills. Furthermore, Gap26 treatment reduced Cx43 expression and astrocytosis after HI insult, suggesting Cx43 could be considered a therapeutic target ischemic or hypoxic disease treatment (Li et al., 2015). This hypothesis is further supported by recent findings in TBI that have shown increased levels of phosphorylated Cx43 raised exosome marker expression and release, which can be activated as protective mechanisms, improving LTP inhibited in TBI (Chen et al., 2018).

## Final Considerations

During neural activity interglial communication regulates neurotransmitter and K^+^ buffering by potentiating astrocytic syncytium function. This global integration by astrocytes is essential for functional and metabolic coupling of astrocytes and neurons. Under pathological conditions; although there is an increase in Cxs expression that can stimulate the syncytium ensemble, this process is impaired by a shift towards HC activity. This alteration is mediated by several pro-inflammatory cytokines (TNF-α and IL-β), Ca^2+^, and metabolic changes that stimulate the probability of pore opening (Retamal et al., 2007; Froger et al., 2010; Kim Y. et al., 2016). This evidence reveals a rise in Cx HCs activity can be a shared phenomenon in neurodegenerative diseases, ischemia and trauma. Additionally, a hallmark in the onset of these diseases could be a decrease in Cx docking required for functional GJs generation. Therefore, search for molecules that can regulate HC pore opening and connexon ensemble can have therapeutic potential.

## Author Contributions

LCM and AVR wrote the article and drew the figures. J-JS wrote the article and SLA conceptualized and wrote the article.

## Conflict of Interest Statement

The authors declare that the research was conducted in the absence of any commercial or financial relationships that could be construed as a potential conflict of interest.
